# Correlation between metabolic syndrome indicators and locomotive syndrome in Chinese geriatric oncology inpatients

**DOI:** 10.3389/fmed.2025.1644170

**Published:** 2025-09-05

**Authors:** Ling Song, Yu-Tong Jing, Ling Li, Yu-Ling Yang, Yun Chen, Xiu-Feng Wu, Ying Chen, Hui Yu, Rui-Rong Wu

**Affiliations:** ^1^Department of Oncology, Affiliated Hospital of Jiangnan University, Wuxi, Jiangsu Province, China; ^2^Wuxi Medical College of Jiangnan University, Lihu Avenue, Wuxi, Jiangsu Province, China; ^3^Department of Pharmacy, Affiliated Hospital of Jiangnan University, Wuxi, Jiangsu Province, China

**Keywords:** locomotive syndrome, metabolic syndrome, aging, geriatric oncology inpatients, correlation

## Abstract

**Objective:**

To investigate the correlation between metabolic syndrome (MS) indicators and locomotive syndrome (LS) in geriatric oncology inpatients.

**Methods:**

This study enrolled 430 geriatric oncology inpatients at risk of LS, admitted to the Department of Oncology at Affiliated Hospital of Jiangnan University from January 2024 and December 2024. Waist circumference, diastolic blood pressure, systolic blood pressure, total cholesterol (TC), triacylglycerols (TG), fasting glucose, high-density lipoprotein cholesterol (HDL-C), and low-density lipoprotein cholesterol (LDL-C) were measured as MS indicators. The Geriatric Locomotive Function Scale (GLFS-25) was used to assess LS. Subjects were classified into two groups: those with LS (322 cases) and those without (87 cases), to analyze the correlation between MS indicators and LS.

**Results:**

409 geriatric oncology inpatients completed the study. One-way linear regression analysis revealed that waist circumference, systolic blood pressure, diastolic blood pressure, TG, and LDL-C were positively correlated with GLFS-25 (*p* < 0.05), while HDL-C was negatively correlated in geriatric oncology inpatients (*p* < 0.05). Logistic regression analysis identified waist, systolic blood pressure, TG, and LDL-C as risk factors for developing LS in geriatric oncology inpatients (*p* < 0.05).

**Conclusion:**

Certain risk factors for MS are associated with increased GLFS-25 scores and the development of LS in geriatric oncology inpatients. Screening for LS is beneficial for the early diagnosis of MS and using LS as a focal point for intervention offers new insights into the comprehensive rehabilitation of geriatric oncology patients.

## 1 Background

Metabolic Syndrome (MS) is a complex clinical syndrome characterized by multiple concurrent pathologies, including abdominal obesity, elevated blood pressure, dyslipidemia, and hyperuricemia (1). These pathologic states constitute the core of MS, which stems from the pathophysiology of diverse metabolic disturbances. These disturbances are significant risk factors for promoting atherosclerosis, potentially leading to the progression of cardiovascular and cerebrovascular diseases, thus posing a serious threat to patients’ quality of life and life expectancy (2). Currently, MS represents a significant public health challenge in China, contributing to substantial medical and economic burdens and increasing socio-economic pressures by impacting workforce health and social productivity (3). Despite the lack of universally recognized treatment methods for MS, increasing research suggests that interventions aimed at improving musculoskeletal function (MF) in MS patients are both cost-effective and beneficial, and thus merit broader implementation (4).

In 2020, there were 19.29 million new cases of tumors globally and 4.57 million in China, predominantly among individuals aged 60–79 (5, 6). This demographic is expected to grow substantially in the coming decades due to changes in social life patterns and advances in medical technology (7, 8). Recent evidence indicates that hormonal changes associated with MS may increase cancer susceptibility through mechanisms, such as inflammatory responses, oxidative stress, growth hormone dysregulation, and vascular endothelial growth factor production (9, 10). Concurrently, as the survival of cancer patients increases, the prevalence of tumors coexisting with other chronic diseases is rising. MS significantly influences the relationship between cancer treatment and the morbidity and mortality associated with tumor-related chronic diseases, while also elevating the risk of other chronic conditions (11).

The current aim of geriatric oncology treatment is not only to improve prognosis but also to enhance the quality of life (QoL) (12). A crucial element in enhancing QoL involves maintaining and improving patients’ MF to support basic activities of daily living (ADL), thereby maximizing their ability to live independently and reducing the caregiving burden on family and society (13).

Locomotive Syndrome (LS), originally proposed by the Japanese Orthopaedic Association (JOA), is a high-risk condition characterized by difficulties in standing, walking, and other movements due to the weakening or impairment of the locomotor organs (bones, joints, muscles, and nerves, etc.). This condition leads to limited MF, increased care needs, and the potential for becoming bedridden (13, 14). Research indicates that LS is strongly associated with adverse outcomes such as decreased ADLs, fractures, and increased mortality (13, 15). Timely identification and intervention are crucial for successful rehabilitation (16, 17). LS focuses more on assessing an individual’s overall MF than on specific symptoms such as sarcopenia and debilitation (18, 19). The deterioration of muscles, bones, and other motor organs often progresses slowly and is difficult to detect (20). LS acts as a sensitive monitor of motor dysfunction and can provide early warnings of MF decline in patients who have not yet shown significant pathological changes (17). Studies have demonstrated that LS-1 and LS-2 states are reversible, and effective physical interventions at these stages can maintain or even improve an individual’s MF and ADL before irreversible physical impairment occurs (20).

LS and MS are both associated with aging (2, 14). Understanding the relationship between LS and MS in geriatric oncology patients facilitates the use of targeted interventions based on LS, leading to timely warnings and interventions that can prevent the regression of rehabilitation outcomes in these patients toward severity. Therefore, this study analyzed the occurrence of MS and LS in hospitalized elderly patients to provide new insights for the rehabilitation of elderly tumor patients.

## 2 Materials and methods

This investigation utilized a single-center, cross-sectional study design. Participants voluntarily joined the study and provided written informed consent. The Ethics Committee of the Affiliated Hospital of Jiangnan University approved the study (no. LS2023101), registered in the Chinese Clinical Trial Registry (no. ChiCTR2400079958) on 17/01/2024. The study adhered to the principles outlined in the Declaration of Helsinki.

### 2.1 Subjects

430 geriatric oncology inpatients at risk of LS were recruited from the Department of Oncology at the Affiliated Hospital of Jiangnan University between January 2024 and December 2024.

#### 2.1.1 Inclusion criteria

① Patients must have had a definitive cancer diagnosis; ② Age > 60 years old; ③ Stable vital signs; ④ Cognitive clarity and coherent responsiveness; ⑤ Capability for autonomous self-care; ⑥ Absence of any MF disorder attributable to primary orthopedic diseases; ⑦ Provided informed consent and voluntarily participated in the study.

#### 2.1.2 Exclusion criteria

① Patients with cognitive impairment or unconsciousness; ② Diagnosis of any incapacitating ailment contraindicating physical activity.

### 2.2 Measurements

A cross-sectional questionnaire, developed after a review of relevant literature on LS from databases such as Web of Science, Embase et al. and designed with input from clinical experts, was employed. This case report form included five parts:

#### 2.2.1 General information

This section gathered data on the subjects’ gender, age, height, weight, self-care ability, marital status, initial tumor type, disease duration, metastatic recurrence, radiotherapy, and comorbidities (including hypertension, diabetes mellitus, dyslipidemia, stroke and chronic respiratory diseases).

#### 2.2.2 LS

The presence of LS was assessed using the GLFS-25, a specialized screening scale recommended by the JOA (21). Validated by Chinese scholars, the GLFS-25 is deemed suitable for Chinese elderly tumor patients (22, 23). It consists of 25 items across four dimensions: physical pain, ADL, social activities, and mental health status. It employs a Likert 5-point scale, ranging from 0 (“no difficulty”) to 4 (“very difficult”), with a total score between 0 and 100. A score of ≥16 indicates LS; higher scores reflect worse MF and more severe LS. The GLFS-25 has demonstrated good reliability and validity: Cronbach’s *α* is 0.961, and retest correlation coefficients range from 0.712 to 0.924 (21). Subjects were categorized into an LS group (322 cases) and a non-LS group (87 cases) based on the presence of LS.

#### 2.2.3 MS

Diagnosis of MS was based on the criteria set forth in the Chinese Guidelines for the Prevention and Control of Type 2 Diabetes Mellitus (2022 edition) (24): (1) Body mass index (BMI) ≥ 25 kg/m^2^; (2) Fasting plasma glucose (FPG) ≥ 6.1 mmol/L or diagnosed and treated diabetes; (3) Hypertension with systolic/diastolic blood pressure ≥140/90 mmHg or diagnosed and treated hypertension; (4) Dyslipidemia with fasting blood triglycerides ≥1.7 mmol/L and/or fasting blood HDL-C < 0.9 mmol/L for men and <1.0 mmol/L for women. MS is diagnosed when three or all four criteria are met. This study collected data on waist circumference, diastolic blood pressure, systolic blood pressure, total cholesterol (TC), triacylglycerols (TG), fasting glucose, high-density lipoprotein cholesterol (HDL-C), and low-density lipoprotein cholesterol (LDL-C) from the study population.

### 2.3 Statistical methods

SPSS 25.0 was applied for statistical analysis, the measurement data were expressed as mean ± standard deviation (Mean ± SD), the count data were expressed as frequency or frequency, *t* test was used to compare the means between the two groups, and *χ*^2^ test or consecutively corrected *χ*^2^ test was used to compare the rates. Linear regression analysis was conducted with GLFS-25 scores as the dependent variable and MS-related indices as independent variables. Logistic regression analysis was conducted with MS-related indices as independent variables, and LS as the dependent variable (Yes = 1, No = 0), using the backward method (Entry = 0.05, Removal = 0.10). The difference was considered statistically significant at *p* < 0.05.

## 3 Results

### 3.1 Prevalence of LS and MS

**Figure 1:**
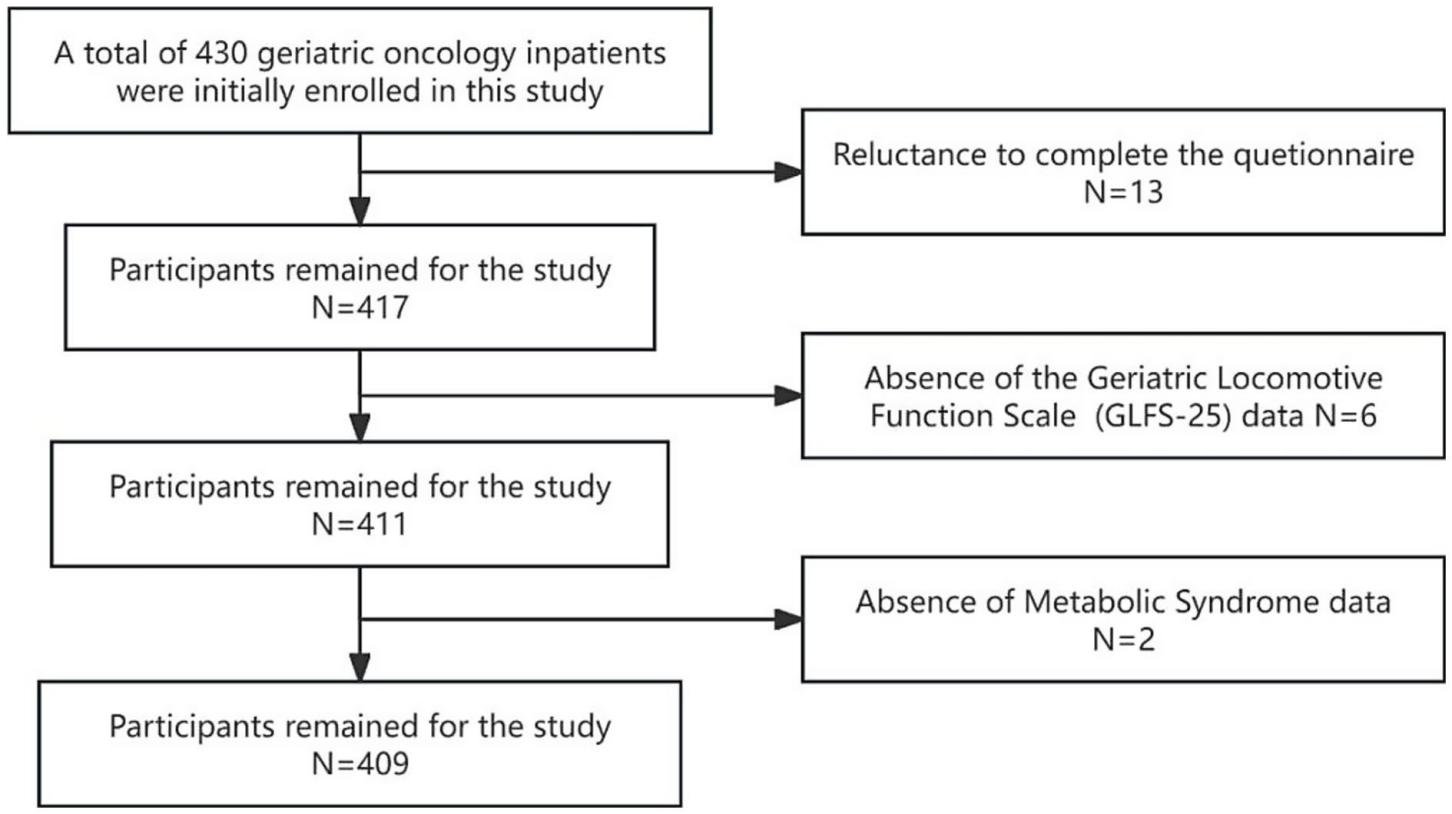
Flow chart of study participants.

**Figure 2:**
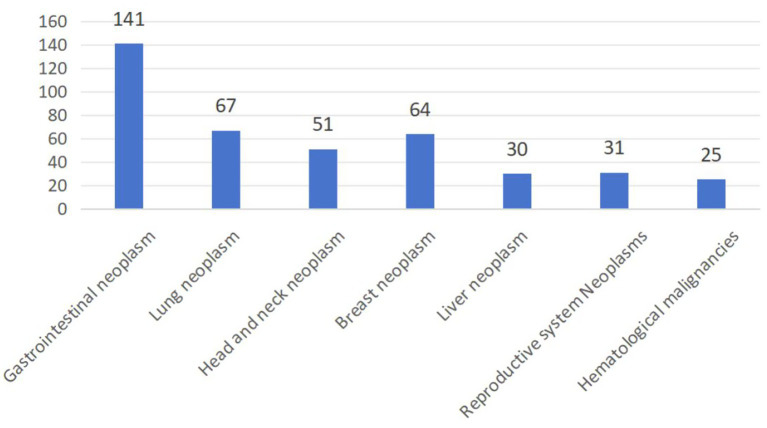
The origin cancer of 409 geriatric cancer inpatients.

A total of 409 geriatric oncology inpatients completed the survey, achieving a questionnaire recovery rate of 95.1% (Figure 1). 13 participants withdrew due to privacy concerns, 6 failed to complete the GLFS-25 questionnaire, and 2 declined to disclose MS-related information. The diagnostic distribution was as follows: gastrointestinal neoplasm comprised 34.5% (141 cases); lung neoplasm 16.4% (67 cases); head and neck neoplasm 12.5% (51 cases); breast neoplasm 15.6% (64 cases); liver neoplasm 7.3% (30 cases); neoplasms of the reproductive system 7.6% (31 cases); and hematological malignancies 6.1% (25 cases) (Figure 2). 65.2% (267 cases) of the participants did not present any co-morbid chronic conditions, while 26.7% (109 cases) had one to two additional chronic conditions, and 8.1% (33 cases) reported three or more. The prevalence of LS was 78.7% (322/409), and the prevalence of MS was 42.8% (175/409). The prevalence of MS was higher in the LS group at 45.3% (146/322) compared to 33.3% (29/87) in the non-LS group, with this difference being statistically significant (*p* < 0.05).

### 3.2 Clinical information

Patients in the LS group exhibited higher measures of gender, body mass, waist circumference, systolic and diastolic blood pressure, TG, and LDL-C compared to those in the non-LS group; these differences were statistically significant (*p* < 0.05). Differences in other clinical indicators were not statistically significant (*p* > 0.05). Details are provided in Table 1.

**Table 1:** Comparison of general information and metabolic syndrome indicators in patients with different locomotive syndrome statuses.

Variables	LS group (*n* = 322)	Non-LS group (*n* = 87)	*t*/*χ*^2^	*P*
Age (years)	74.81 ± 7.69	73.13 ± 7.32	1.289	0.196
Gender (male/female, *N*)	155/167	42/45	0.001	0.964
Height (cm)	168.33 ± 9.03	167.47 ± 8.76	1.179	0.213
Weight (kg)	70.92 ± 8.87	68.67 ± 9.72	2.384	0.023^∗^
BMI (kg/m^2^)	24.83 ± 3.86	24.24 ± 4.76	1.279	0.245
Waist (cm)	95.02 ± 12.65	81.58 ± 14.56	5.083	0.000^∗^
Systolic blood pressure (mmHg)	136.65 ± 18.78	124.51 ± 16.43	3.591	0.000^∗^
Diastolic blood pressure (mmHg)	84.69 ± 13.78	79.23 ± 8.24	2.894	0.000^∗^
Fasting glucose (mmol/L)	5.51 ± 1.39	5.32 ± 1.32	0.989	0.365
TC (mmol/L)	4.84 ± 1.07	4.71 ± 1.08	1.075	0.246
TG (mmol/L)	3.21 ± 1.76	2.43 ± 1.55	2.886	0.004^∗^
HDL-C (mmol/L)	1.53 ± 0.56	1.57 ± 0.50	−1.109	0.283
LDL-C (mmol/L)	3.17 ± 0.92	2.61 ± 0.87	3.112	0.003^∗^

^∗^: *P* < 0.05.

### 3.3 Linear regression analysis of MS indicators and GLFS-25 score

Linear regression analysis was conducted with GLFS-25 scores as the dependent variable and MS-related indices as independent variables. Results indicated that waist circumference, systolic and diastolic blood pressure, TG, and LDL-C were positively correlated with GLFS-25 scores (*p* < 0.05), while HDL-C was negatively correlated (*p* < 0.05). Details are provided in Table 2.

**Table 2:** Linear regression analysis of metabolic syndrome indicators and GLFS-25 scores.

Variables	*B*	95% *CI*	*P*
Waist	0.042	0.028 ~ 0.055	0.000^∗^
Systolic blood pressure	0.045	0.024 ~ 0.057	0.000^∗^
Diastolic blood pressure	0.061	0.043 ~ 0.080	0.000^∗^
Fasting glucose	0.166	0.082 ~ 0.335	0.181
TC	-0.043	−0.298 ~ 0.219	0.769
TG	0.411	0.232 ~ 0.531	0.000^∗^
HDL-C	-0.621	-1.154 ~ 0.049	0.029^∗^
LDL-C	0.439	0.151 ~ −0.732	0.003^∗^

^∗^: *P* < 0.05.

### 3.4 Logistic regression analysis of MS indicators and LS

Logistic regression analysis was conducted with age, gender, waist circumference, systolic blood pressure, diastolic blood pressure, fasting blood glucose, TC, TG, HDL-C, and LDL-C as independent variables, and LS as the dependent variable (Yes = 1, No = 0), using the backward method (Entry = 0.05, Removal = 0.10). The assignment of values to the independent variables is detailed in Table 3. The multifactorial analysis identified waist circumference, systolic blood pressure, TG, and LDL-C as independent risk factors for the development of LS (*p* < 0.05). Further details are provided in Table 4.

**Table 3:** Independent variable assignment.

Independent variable	Assignment
Age, waist, systolic blood pressure, diastolic blood pressure, fasting glucose, TC, TG, HDL-C, LDL-C	Numerical value
Gender	Male = 1; Female = 0;

**Table 4:** Logistic regression analysis of metabolic syndrome indicators and locomotive syndrome.

Variables	*B*	*P*	OR	95% CI
Age	0.030	0.203	1.033	0.975 ~ 1.078
Gender	−0.074	0.865	0.931	0.453 ~ 1.832
Waist	0.058	0.000^∗^	1.057	1.021 ~ 1.082
Systolic blood pressure	0.039	0.001^∗^	1.042	1.021 ~ 1.064
Diastolic blood pressure	0.054	0.062	1.056	1.021 ~ 1.089
Gasting glucose	0.129	0.334	1.142	0.868 ~ 1.468
TC	0.179	0.276	1.187	0.859 ~ 1.659
TG	0.293	0.004^∗^	1.338	1.097 ~ 1.621
HDL-C	−0.393	0.274	0.668	0.345 ~ 1.334
LDL-C	0.607	0.002^∗^	1.841	1.229 ~ 2.743

^∗^: *P* < 0.05.

## 4 Discussion

In the current Chinese context, medical resources are primarily allocated to emergency oncology treatments, often at the expense of oncology rehabilitation, which remains underfunded. There is a pressing need for a highly sensitive, straightforward, and practical MF test indicator to quickly ascertain the health status of elderly tumor patients (25). Most existing MF assessment methods involve numerous specialized instruments and personnel, making the process complex and time-consuming. Many patients are hesitant to dedicate significant time and resources to MF testing, which may lead to unrecognized worsening of MF and missed opportunities for timely recovery, adversely affecting their prognosis (26). LS presents a viable solution by serving as an efficient and accessible indicator for detecting MF impairments. By providing early warnings of irreversible MF damage, LS can prevent severe MF injuries and their associated sequelae, thereby saving considerable time, money, and caregiving resources. Thus, LS was employed as a measure of MF in this study.

The results of the study indicated that the prevalence of MS among geriatric oncology inpatients was significantly higher in the LS group at 45.3% (146/322) compared to 33.3% (29/87) in the non-LS group. Furthermore, MS-related indicators (waist circumference, systolic blood pressure, diastolic blood pressure, TG, LDL-C, and HDL-C) were significantly correlated with GLFS-25 scores (*p* < 0.05). Waist circumference, systolic blood pressure, diastolic blood pressure, TG, and LDL-C were identified as risk factors for GLFS-25, while HDL-C acted as a protective factor. This suggests a positive association between MS and the occurrence of LS.

This may be attributed to the skeletal system and skeletal muscle, which constitute approximately 60% of body weight and are integral to adult musculoskeletal function (MF), influencing LS status (27). They also function as the body’s largest endocrine organ. Skeletal muscle synthesizes and secretes various cytokines, regulatory peptides, adipokines, growth factors, and proteins specific to bone and muscle regulation (28). These paracrine/autocrine factors are involved in the pathogenesis of central obesity, insulin resistance, dyslipidemia, hypertension, and microalbuminuria, forming a crucial pathophysiological foundation for MS (27).

Currently, public awareness and understanding of LS status in elderly tumor patients are inadequate, with limited knowledge on its scientific prevention and treatment. This study demonstrates a significant correlation between various MS indicators and LS, suggesting that addressing LS could illuminate new pathways for the rehabilitation of elderly tumor patients. Enhancing early screening and intervention for LS could also improve public knowledge and scientific understanding of LS.

JAMA suggests that physical activity serves as an effective non-pharmacological therapeutic approach, improving the overall physiological functions of patients with LS by regulating insulin resistance, adipocytokines, and lipid metabolism, thereby facilitating the prevention and treatment of MS. Developing tailored exercise prescriptions for LS also enhances the rehabilitation of geriatric oncology patients (17, 29). It is recommended that geriatric oncology patients select the appropriate type, intensity, and duration of exercise based on age, health status, and personal preference, utilizing heart rate monitoring as a guideline. Special attention should be given to their physical tolerance, adjusting exercise intensity to ensure safety and effectiveness, and to optimize metabolic indices and LS status continually. Exercise parameters such as intensity, duration, and frequency should adhere to the principles of individualization and gradual progression. If feasible, a professional cardiopulmonary exercise test may be performed prior to developing an exercise plan to scientifically tailor the exercise regimen.

## 5 Conclusion

In conclusion, our findings demonstrate an association between certain risk factors for MS and elevated GLFS-25 scores, suggesting a potential link with LS in geriatric oncology inpatients. While this cross-sectional study identifies these relationships, further longitudinal research is needed to determine whether LS screening could aid in early MS detection. Nevertheless, incorporating LS assessment into clinical evaluation may offer valuable insights for optimizing rehabilitation strategies in this vulnerable population.

## 6 Limitations

This study was limited to hospitalized elderly patients, representing a small sample size that does not reflect the broader population of geriatric oncology patients. Additionally, this study does not establish a causal relationship between abnormal metabolic indices and LS, necessitating further research with larger sample sizes and prospective designs.

## Data Availability

The raw data supporting the conclusions of this article will be made available by the authors, without undue reservation.
